# Supplementary material for: Focal Adhesion Maturation Responsible for Behavioral Changes in Human Corneal Stromal Fibroblasts on Fibrillar Substrates
Source: Int J Mol Sci. 2024 Aug 7;25(16):8601. doi: 10.3390/ijms25168601 (PMC11354758; doi:10.3390/ijms25168601)
Supplement: Supplementary file 1 [file ijms-25-08601-s001.zip › Supplementary Information_Zhurenkov et al 2024_IJMS.pdf]

Supplementary Information for

Focal Adhesion Maturation Responsible for Behavioral Changes in Human Corneal Stromal Fibroblasts on Fibrillar Substrates

*Kirill E. Zhurenkov 1,2,†, Arseniy A. Lobov 1, Natalya B. Bildyug 1, Elga I. Alexander-Sinclair 1, Diana M. Darvish 1, Ekaterina V. Lomert 1, Daria V. Kriger 1, Bozhana R. Zainullina 3, Alina S. Chabina 1, Julia I. Khorolskaya 1,‡, Daria A. Perepletchikova 1, Miralda I. Blinova 1 and Natalia A. Mikhailova 1,**

1 Institute of Cytology Russian Academy of Sciences, St. Petersburg 194064, Russia; kzhu576@aucklanduni.ac.nz (K.E.Z.); arseniylobov@gmail.com (A.A.L.); relapse@yandex.ru (N.B.B.); elga.aleks@gmail.com (E.I.A.-S.); darvishdi@mail.ru (D.M.D.); e.lomert@gmail.com (E.V.L.); daryamalikova@gmail.com (D.V.K.); chabinaalina123@gmail.com (A.S.C.); j.i.khorolskaya@gmail.com (J.I.K.); dasha_perepletch@mail.ru (D.A.P.); mira.blinova@mail.ru (M.I.B.)

2 Department of Cytology and Histology, St. Petersburg State University, St. Petersburg 199032, Russia;

3 Centre for Molecular and Cell Technologies, St. Petersburg State University, St. Petersburg 199032, Russia; zainullinazhana@gmail.com

* Correspondence: natalia.mikhailova@incras.ru

† Current address: Department of Chemical and Materials Engineering, The University of Auckland, Auckland 1010, New Zealand.

‡ Current address: Department of Ophthalmology, University Hospital Erlangen, Friedrich-Alexander-University Erlangen-Nürnberg, D-91054 Erlangen, Germany.

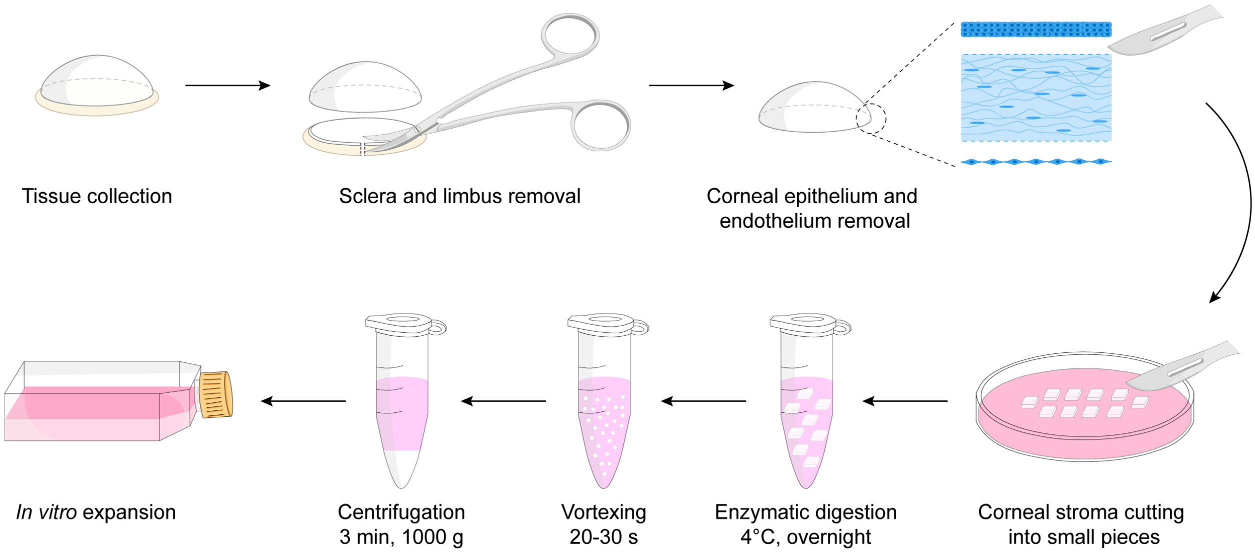

Figure S1. Corneal stromal fibroblasts (CSFs) isolation procedure.

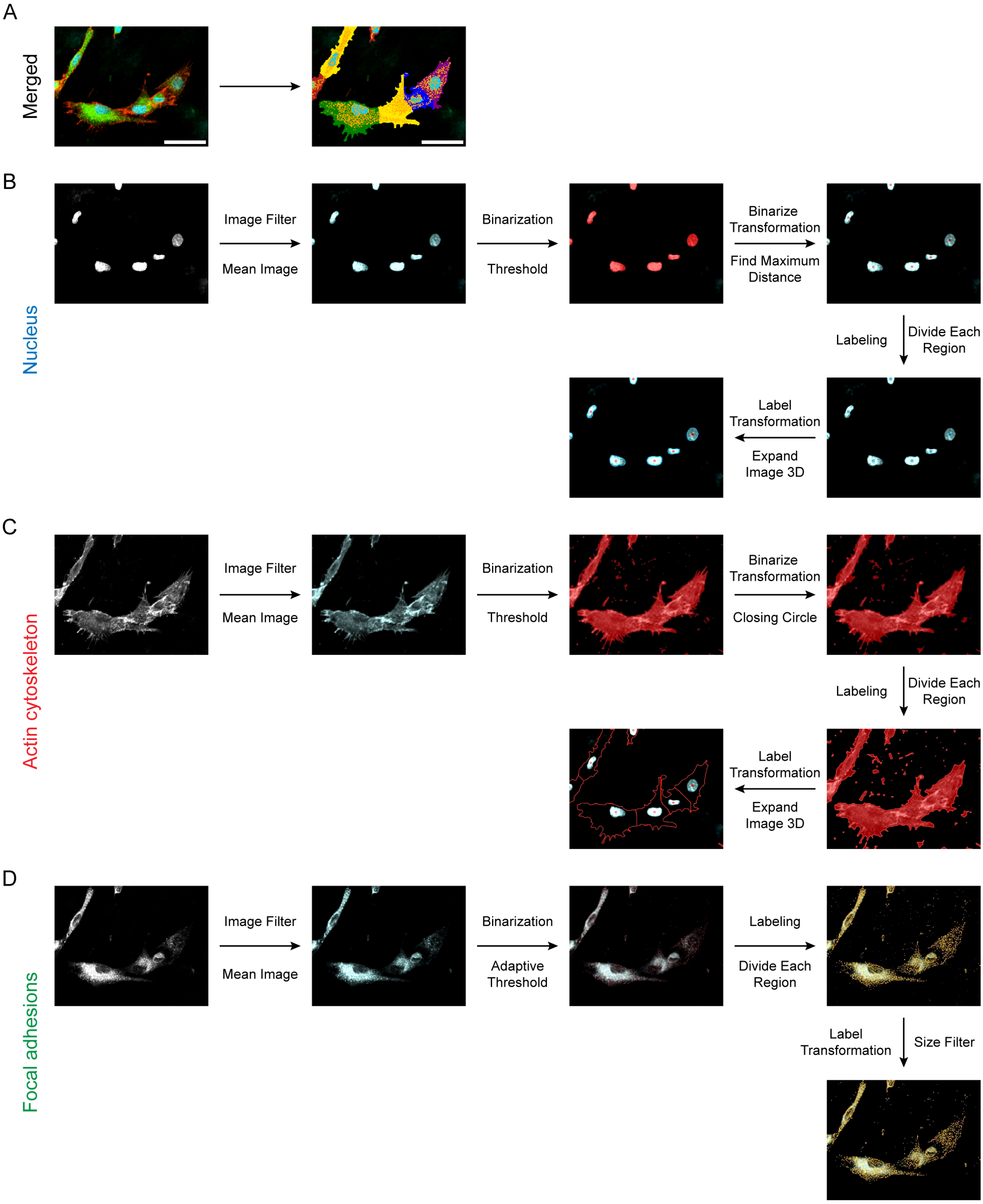

Figure S2. Quantitative immunofluorescence analysis protocol for paxillin. Representative images and steps of quantitative immunofluorescence analysis of CSFs are presented as follows: **(A)** CSFs cultured on a specific substrate for 8 hours; **(B)** staining of DAPI for the nucleus, shown in blue; **(C)** Phalloidin staining for the actin cytoskeleton, depicted in red; and **(D)** paxillin staining for focal adhesions, appearing in green. Scale bars: 50 μm.

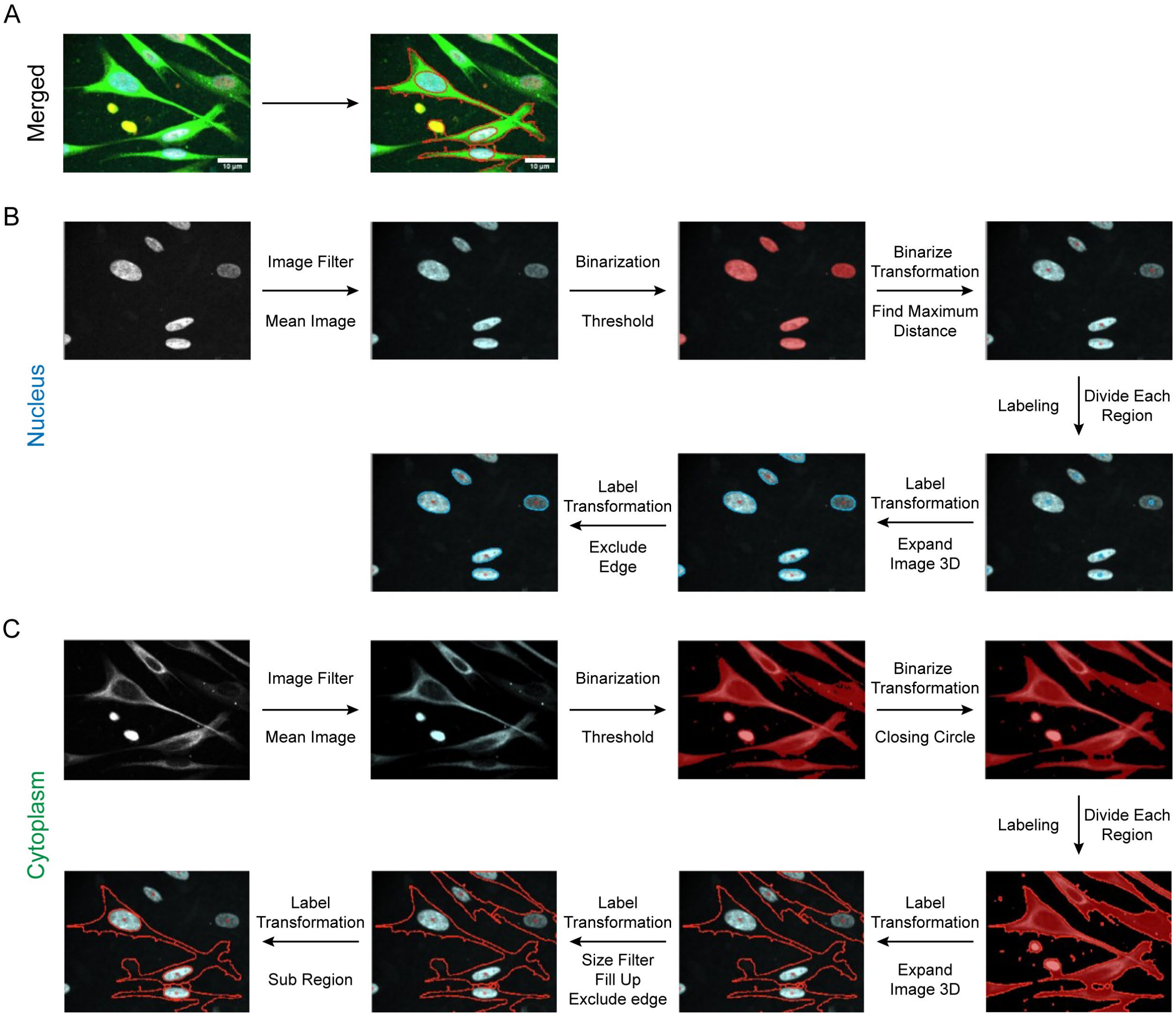

Figure S3. Quantitative immunofluorescence analysis protocol for YAP1. Representative images and steps of quantitative immunofluorescence analysis of CSFs are presented as follows: **(A)** CSFs cultured on a specific substrate for 8 hours; **(B)** staining of DAPI for the nucleus, shown in blue; and **(C)** YAP1 staining, appearing in green. Scale bars: 10 μm.

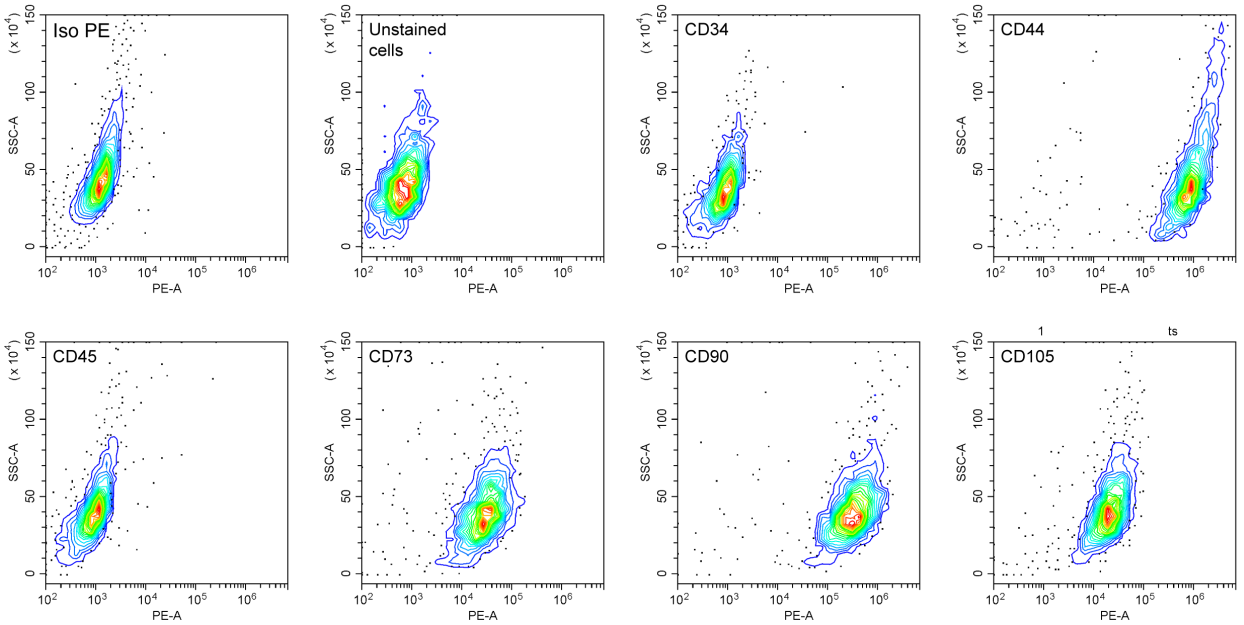

Figure S4. Corneal stromal fibroblasts (CSFs) culture characterization. Features of specific hematopoietic and mesenchymal cluster of differentiation (CD) marker expression in cultured CSFs at passage 6. Measurements were collected on cells from three independent experiments using CSFs from three different donors (n = 3). Iso PE was used as the negative control.

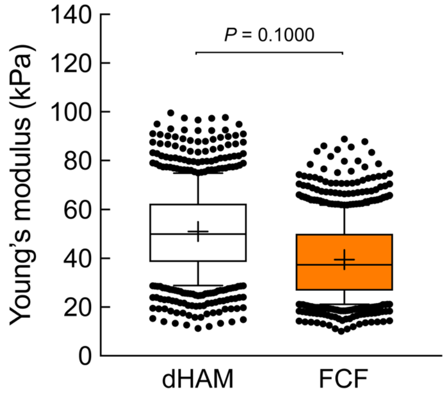

Figure S5. The Young's moduli of dHAM and FCF. Box plots reflect the sample's Young's modulus in kPa. The box indicates the interquartile range (IQR) between the first and the third quartiles, while whiskers denote 1.5 IQR. The plus and the long horizontal line represent the mean and the median, respectively. The data are shown as a distribution from the 10th to the 90th percentile (three samples were analyzed per every condition, representing 300 force curves each). Mann-Whitney statistics is displayed (n = 3; P = 0.1000).

Atomic force microscopy (AFM) was exploited to collect force curves with pre-calibrated spherical (k = 0.07) silicon nitride cantilevers with 5 μm in a diameter spherical borosilicate tip (Novascan, Chicago, IL, USA), using an MFP-3D Origin™ AFM (Asylum Research, Santa Barbara, CA, USA). The deflection sensitivity in HEPES buffer (20 mM HEPES, 120 mM NaCl, 4 mM KCl, 2 mM $CaCl_2$, 22 mM $NaHCO_3$, 1 mM Na_2HPO_4) was determined by fitting the slope of a force-indentation curve performed on a clean silicon wafer (1.0 V trigger and 2 μm s^{-1} indentation velocity) to determine the inverse optical lever sensitivity (invOLS). AFM force-indentation curves were collected in a liquid environment (HEPES buffer) from the samples at a speed of 4 μm s^{-1} and trigger force of 3 nN, with a minimum of 10 × 10 points over a 90 μm^2 area.

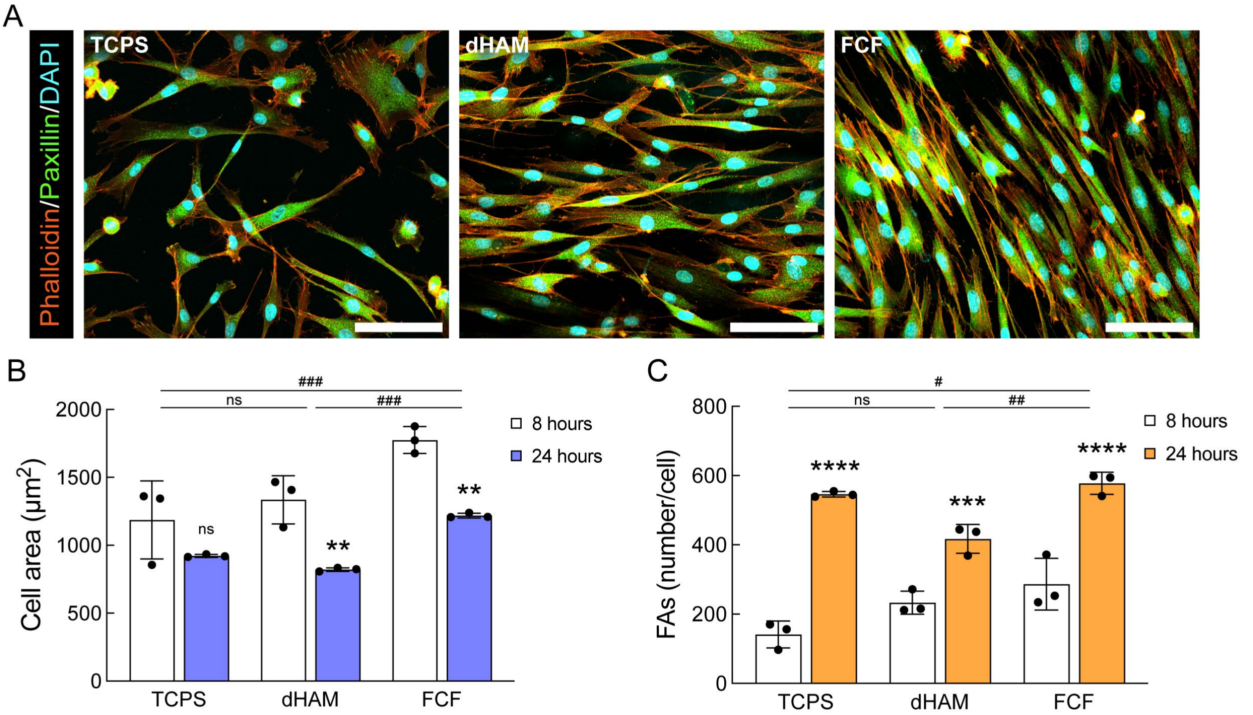

Figure S6. Human corneal stromal fibroblast (CSF) culturing on different substrates during 24 hours. (A) Fluoresce images of CSFs stained for actin cytoskeleton (visualized with Phalloidin-TRITC, red), paxillin (green), and DAPI (blue). Scale bars: 100 μm. (B,C) CSF spreading features on the investigated substrates after 24 hours of culturing. The bar graphs are represented as average ± S.D. and derived from three independent experiments (n = 3; ** corresponds to $p < 0.001$, *** corresponds to $p < 0.0002$, and **** corresponds to $p < 0.0001$ after two-way ANOVA followed by Bonferroni's multiple comparisons post hoc test compared to CSFs cultured for 8 hours). Additional statistical analysis was conducted to compare different substrates (# corresponds to $p < 0.05$, ## corresponds to $p < 0.001$, and ### corresponds to $p < 0.0002$ after two-way ANOVA followed by Bonferroni's multiple comparisons post hoc test). Quantitative immunofluorescence analysis (q-IFA).

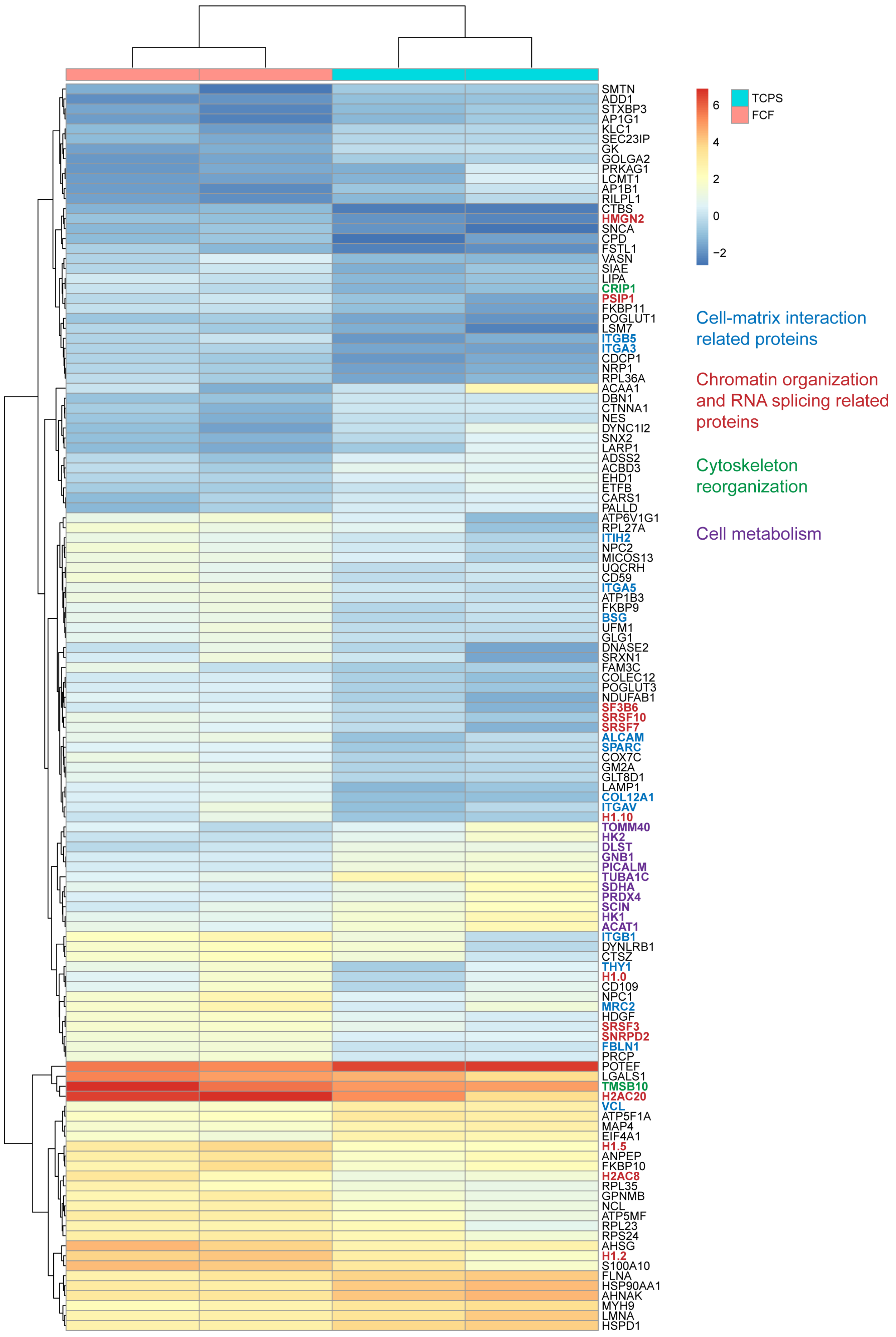

TCPS
FCF
6
4
2
0
−2
SMTN
ADD1
STXBP3
AP1G1
KLC1
SEC23IP
GK
GOLGA2
PRKAG1
LCMT1
AP1B1
RILPL1
CTBS
HMGN2
SNCA
CPD
FSTL1
VASN
SIAE
LIPA
CRIP1
PSIP1
FKBP11
POGLUT1
LSM7
ITGB5
ITGA3
CDCP1
NRP1
RPL36A
ACAA1
DBN1
CTNNA1
NES
DYNC1I2
SNX2
LARP1
ADSS2
ACBD3
EHD1
ETFB
CARS1
PALLD
ATP6V1G1
RPL27A
ITIH2
NPC2
MICOS13
UQCRH
CD59
ITGA5
ATP1B3
FKBP9
BSG
UFM1
GLG1
DNASE2
SRXN1
FAM3C
COLEC12
POGLUT3
NDUFAB1
SF3B6
SRSF10
SRSF7
ALCAM
SPARC
COX7C
GM2A
GLT8D1
LAMP1
COL12A1
ITGAV
H1.10
TOMM40
HK2
DLST
GNB1
PICALM
TUBA1C
SDHA
PRDX4
SCIN
HK1
ACAT1
ITGB1
DYNLRB1
CTSZ
THY1
H1.0
CD109
NPC1
MRC2
HDGF
SRSF3
SNRPD2
FBLN1
PRCP
POTEF
LGALS1
TMSB10
H2AC20
VCL
ATP5F1A
MAP4
EIF4A1
H1.5
ANPEP
FKBP10
H2AC8
RPL35
GPNMB
NCL
ATP5MF
RPL23
RPS24
AHSG
H1.2
S100A10
FLNA
HSP90AA1
AHNAK
MYH9
LMNA
HSPD1
FCF 1
FCF 2
TCPS 1
TCPS 2
Cell-matrix interaction related proteins
Chromatin organization and RNA splicing related proteins
Cytoskeleton reorganization
Cell metabolism

Figure S7. Extended heatmap of top-differentially expressed proteins between human corneal stromal fibroblasts (CSFs) cultured on TCPS and FCF after 8 days.

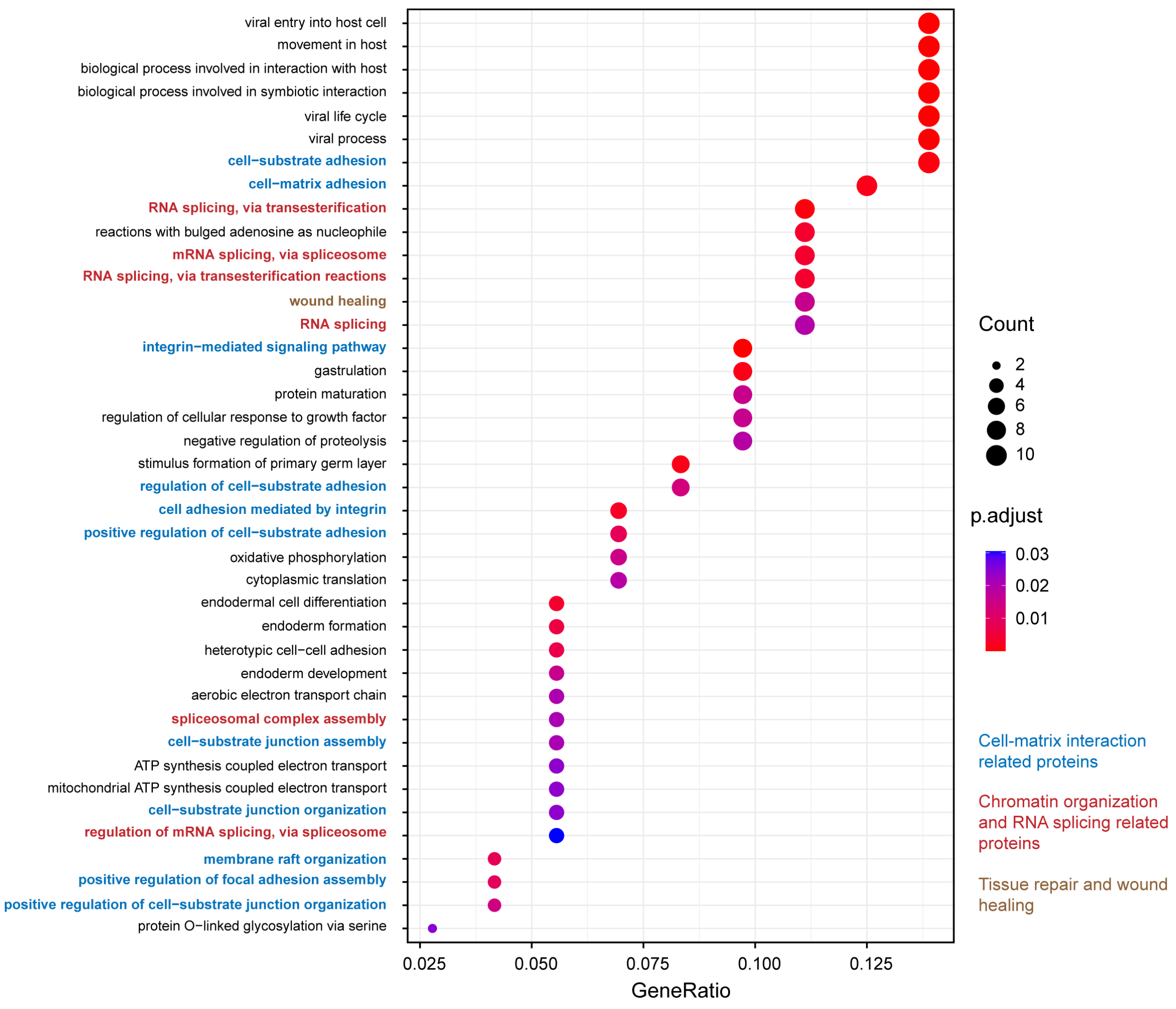

Figure S8. Gene Ontology (GO) biological processes pathway enrichment analysis of up-regulated proteins specific for human corneal stromal fibroblasts (CSFs) cultured on FCF compared to TCPS during 8 days.

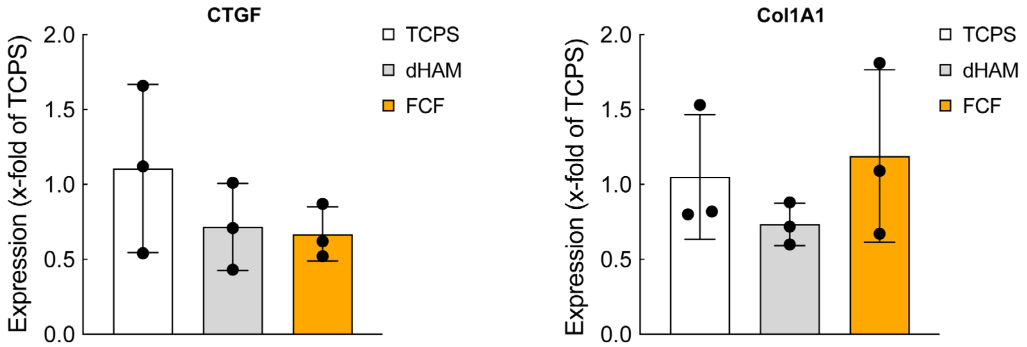

Figure S9. FAK inhibition (pFAKi) down-regulates *CTGF* and *Col1A1* gene expression in human corneal stromal fibroblasts (CSFs) cultured on fibrillar substrates. *CTGF* and *Col1A1* mRNA expression after 2 days of culturing. The bar graphs are represented as average ± S.D. and derived from three independent experiments (n = 3; p = 0.4296 and 0.3355 for *CTGF* and *Col1A1*, respectively, after two-way ANOVA followed by Tukey's multiple comparisons post hoc test compared to TCPS). Quantitative real-time polymerase chain reaction (qRT-PCR). The data is normalized to *GAPDH* and calculated using ΔΔCt method.

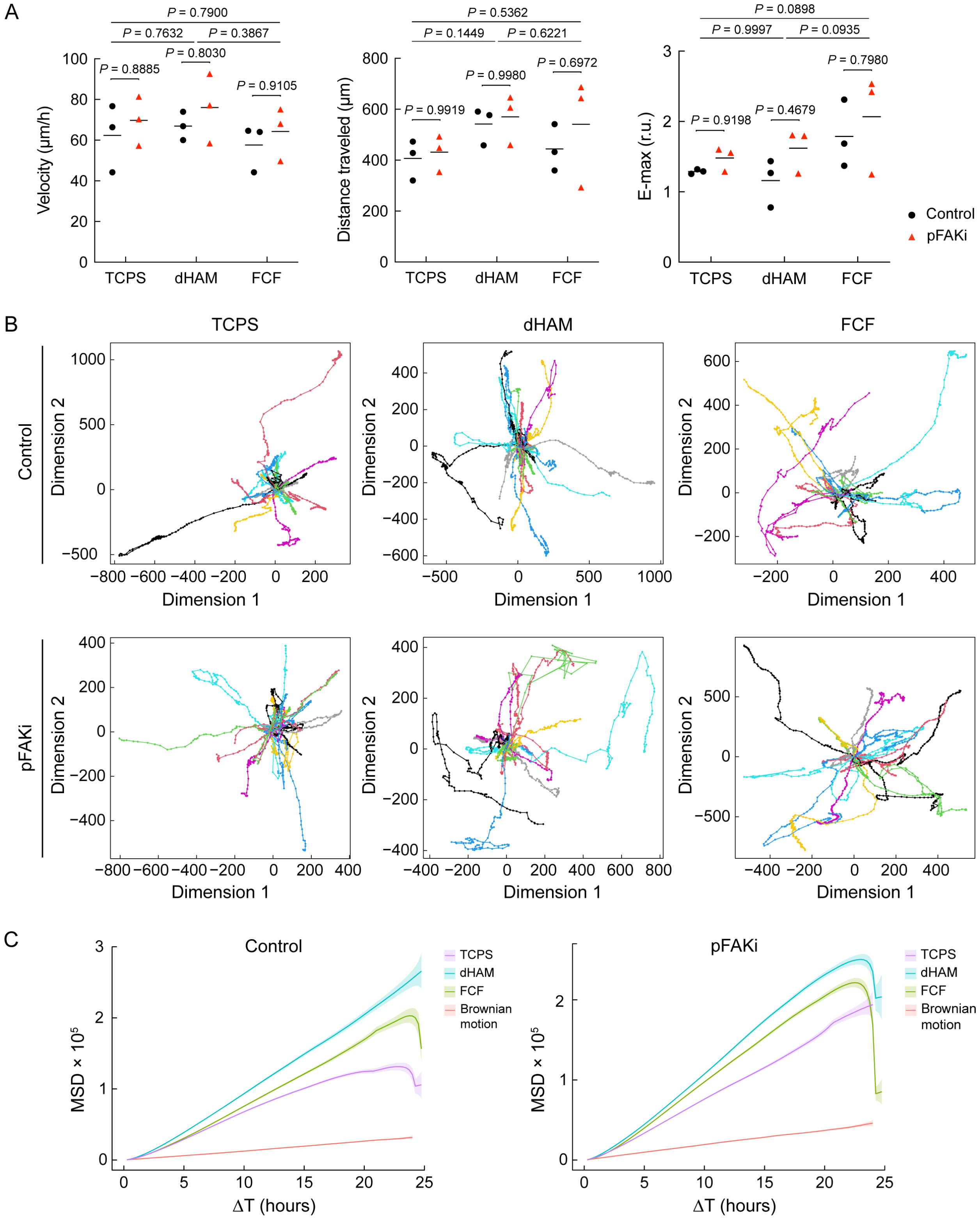

Figure S10. Migration pattern changes in human corneal stromal fibroblasts (CSFs) cultured on fibrillar substrates. **(A)** Average values, representing cell velocity, distance traveled, and the degree of cell movement (E-max), estimated for CSFs cultured on TCPS, dHAM, and FCF during 24 hours (n = 3 with 150 cells analyzed per each biological repeat; displayed P–values are derived from two-way ANOVA followed by Šidák's multiple comparisons post hoc test for the comparison

between control and pFAKi groups and Tukey's multiple comparisons post hoc test for the comparison between various substrates). **(B)** KC migration patterns after culturing on TCPS, dHAM, and FCF during 24 hours with or without pFAKi. Normalized data (n = 20). **(C)** Mean square displacement (MSD) of CSFs cultured on different substrates during 24 hours with or without pFAKi.

Table S1. Antibody set used for flow cytometry.

Antibody	Manufacturer	Identifier
anti-CD34	BD Biosciences, Franklin Lakes, NJ, USA	Cat# 555822
anti-CD44	BD Biosciences, Franklin Lakes, NJ, USA	Cat# 550989
anti-CD45	BD Biosciences, Franklin Lakes, NJ, USA	Cat# 561866
anti-CD73	BD Biosciences, Franklin Lakes, NJ, USA	Cat# 550257
anti-CD90	BD Biosciences, Franklin Lakes, NJ, USA	Cat# 555596
anti-CD105	BD Biosciences, Franklin Lakes, NJ, USA	Cat# 560839
Iso PE	BD Biosciences, Franklin Lakes, NJ, USA	Cat# 554680

CD, cluster of differentiation; PE, phycoerythrin.

Table S2. Antibody set used for immunofluorescence and Western blot analyses.

**Antibody**	**Manufacturer**	**Identifier**
anti-phospho-FAK	Cell Signaling, Danvers, MA, USA	Cat# 3283
anti-FAK	Cell Signaling, Danvers, MA, USA	Cat# 3285
anti-GAPDH	Sigma-Aldrich, St. Louis, MO, USA	Cat# G9545
anti-Paxillin	Abcam, Cambridge, UK	Cat# ab32084
anti-YAP1	Abcam, Cambridge, UK	Cat# ab205270
Goat anti-Mouse IgG H&L	Abcam, Cambridge, UK	Cat# ab150114
Goat anti-Rabbit IgG H&L	Abcam, Cambridge, UK	Cat# ab150077
Pierce® Goat anti-Rabbit IgG H&L, peroxidase conjugated	Pierce Biotechnology, Rockford, IL, USA	Cat# 31460

Table S3. Primer set used for quantitative real-time polymerase chain reaction (qRT-PCR).

Gene	Forward	Reverse	T_m, ℃
PTK2 (FAK)	AAGGTGTACGAGAATGTGACGG	AAGCCGACTTCCTTCACCATAG	60.2
YAP1	TGACGACCAATAGCTCAGATCC	TCATGCTTAGTCCACTGTCTGT	59.4
ALDH3A1	GTTCATCAACCAGCGTGAGAAG	CCACTGGATGTCTCTGCAATCA	60.1
ALDH1A1	ACTTACCTGTCCTACTCACCGA	CTTGCCACTCACTGAATCATGC	60.1
SNAI1	CTCTTTCCTCGTCAGGAAGC	GGCTGCTGGAAGGTAAACTC	58.3
SLUG	TCCAGACCCTGGTTGCTTCA	GAATGGGTCTGCAGATGAGCC	61.1
TWIST1	AGCAGGGCCGGAGACCTAGAT	GCCCCACGCCCTGTTTCTTTGA	65.2
ACTA2	GTTACTACTGCTGAGCGTGAG	CAGGCAACTCGTAACTCTTC	57.3
CCN2 (CTGF)	CCTATTCTGTCACTTCGGCTCC	GTACACCGTACCACCGAAGATG	60.6
Col1A	GACCTAAAGGTGCTGCTGGAG	CTTGTTCACCTCTCTCGCCA	60.2
GAPDH	CAAGGTCATCCATGACAACTTTG	GTCCACCACCCTGTTGCTGTAG	60.6

Table S4. Flow cytometry profile of human corneal stromal fibroblasts (CSFs).

Antigen	Positively stained CSFs, %	Specificity
CD34	0.63% ± 0.46	Hemathopoiethic cells
CD44	99.04% ± 0.61	Mesenchymal cells
CD45	1.33% ± 0.54	Hemathopoiethic cells
CD73	99.03% ± 0.15	Mesenchymal cells
CD90	99.42% ± 0.42	Mesenchymal cells
CD105	99.58% ± 0.21	Mesenchymal cells
Iso PE	0.37% ± 0.43	Negative control

Data are presented as the ratio of the number of cells expressing the antigen to the total number of counted cells and as the means ± S.D. (n=3).
